# An ELISA based on soluble egg antigens for the serodiagnosis of animal schistosomiasis turkestanica

**DOI:** 10.1371/journal.pone.0228184

**Published:** 2020-01-29

**Authors:** Rongyi Ji, Yuanxi Shen, Bin Shi, Hao Li, Wenqiang Tang, Chenyang Xia, Ke Lu, Danqu Lamu, Yang Hong, Xueqiang Sun, Jianzhi Liu, Lanqi Zhang, Chuangang Zhu

**Affiliations:** 1 Key Laboratory of Animal Parasitology, Ministry of Agriculture of China, Shanghai Veterinary Research Institute, Chinese Academy of Agricultural Sciences, Min hang District, Shanghai, China; 2 Jiangsu Co-innovation Center for Prevention and Control of Important Animal Infectious Diseases and Zoonoses, Yangzhou, China; 3 Institute of Animal Science, Tibet Academy of Agricultural and Animal Husbandry Science, Tibet Lhasa, China; 4 China Animal Health and Epidemiology Center, Qingdao, China; Academic Medical Centre, NETHERLANDS

## Abstract

**Background:**

The existing diagnostic techniques for detecting schistosomiasis turkestanica, such as aetiological assays, identify infection by parasitic worms via the incubation of miracidia from faeces or observing eggs under microscopy. However, they are limited in the diagnosis of low-grade and prepatent infections, which lead to a high misdetection rates. Therefore, a new method for parasite diagnosis with increased sensitivity is urgently needed.

**Methods:**

Goats in Nimu County (Tibet, China) infected with *Schistosoma turkestanicum* in an epidemic area were selected according positivity for the infection by faecal examination. Adult worms were collected, eggs were extracted by the sodium hydroxide (NaOH) erosion method, and soluble worm antigen preparation (SWAP) and soluble egg antigen (SEA) were isolated. The best coating concentration of the antigens and the best degree of dilution for serum were determined by square array experiments, and the optimal blocking solution and serum diluents were selected. The specificity, sensitivity and crossover of the ELISA method were determined using 48 samples of goat sera positive for *S*. *turkestanicum*, 100 samples of goat sera negative for *S*. *turkestanicum*, and 54 samples of buffalo sera positive for *S*. *japonicum*. Serological assays were established with samples from goats naturally grazed in a rural area of Nimu County, Tibet Province, by using the indirect ELISA method for the diagnosis of schistosomiasis, and faeces were collected for miracidia hatching. The sensitivity of the two detection methods was compared.

**Results:**

Eggs of *S*. *turkestanicum* were distributed in the host duodenum and small intestine. Eggs in the host intestinal wall were extracted by the NaOH erosion method, which provided intact eggs with reduced impurities. The testing results obtained by isolating SEA were more stable than those obtained by using SWAP and less affected by the coating concentration and serum dilution. Additionally, the value of positive serum/negative (P/N) serum for SEA was much higher than that for SWAP. The optimal coating concentration of SEA was 0.5 μg/ml, and the optimal serum dilution was 1:100. The specificity and sensitivity of the indirect ELISA based on SEA (*S*. *turkestanicum*) were both 100%, and no cross-reactivity was found with schistosomiasis japonica. An epidemiological survey of goats in naturally infected areas showed that the prevalence rate of schistosomiasis turkestanica was 93%, and the infection rate increased with the ages of the goats.

**Conclusion:**

We aimed to develop a sensitive method to utilize in the mass field screening of livestock. As a diagnostic antigen, SEA (*S*. *turkestanicum*) was more suitable for serological testing than SWAP (*S*. *turkestanicum*). The indirect ELISA using SEA (*S*. *turkestanicum*) exhibited good sensitivity, specificity and no cross-reactivity with schistosomiasis japonica. The degree of infectivity and prevalence of *S*. *turkestanicum* infection in endemic areas are serious and should be a focus of concern among local departments.

## Introduction

Schistosomiasis is distributed widely around the world, including in India, Mongolia, Iraq, Kazakhstan and European region, such as Russia and France[1]. Dutt&Srivastava(1955) considered members of the schistosomatid genus Orientobilharzia were parasites of mammals. Five species and one variety had been described and included in the genus. In 2012, Jitka A believed the four valid species previously accommodated in Orientobilharzia should transferred to Schistosoma in light with some molecular and morphological evidence. List of species previously assigned to Orientobilharzia (and their synonyms) and then transferred to Schistosoma, for instance, *Schistosoma bomfordi* (Montgomery, 1906),*Schistosoma dattai* (Dutt & Srivastava, 1952), *Schistosoma harinasutai* (Kruatrachue, Bhaibulaya & Harinasuta, 1965) and *Schistosoma turkestanicum* (Skrjabin, 1913)[2].Schistosomiasis turkestanica is caused by *Schistosoma turkestanicum*, which belongs to platyhelminthes: Trematoda: Digenea: Schistosomatidae[3]. *S*. *turkestanicum* was first found in the portal vein branches of cattle in Russian Turkestan[4]. In China, the disease is endemic in more than 20 provinces, municipality and autonomous regions, especially in north-east, north-west, and south-west China and Inner Mongolia[5].

The development of *S*. *turkestanicum* requires 2–3 hosts: the definitive host, the intermediate host and the reservoir host. Buffaloes, goats and other domestic animals are the major definitive hosts of *S*. *turkestanicum*. According to an epidemiological survey conducted in Mongolia and Shanxi, Hunan and Heilongjiang provinces in China, the infection rates in buffaloes and goats were 66%-100% and 83%-100%, respectively[6]. The maximum parasitic burden in cattle and sheep was 58940 and 53210 worms, respectively[7]. After the penetration of cercariae into the skin of the reservoir host, they eventually develop into adults and lay eggs in the host vessels. The eggs enter the intestine from the blood vessels and hatch outside of the body in the faeces. *S*. *turkestanicum* can cause high mortality in buffaloes and goats and seriously impair the development of animal husbandry[8]. Moreover, *S*. *turkestanicum* can also infect humans and induce cercarial dermatitis in people[9]. Inflammation of the skin occurs after contact with contaminated water; itching and discomfort then appear, and urticaria and secondary pustules may occur as a result of scratching. Therefore, schistosomiasis turkestanica is a serious zoonotic parasitic disease[4,10–13].

The common diagnostic methods for schistosomiasis include direct aetiological observations (parasite egg detection and miracidia hatching) and serological techniques (antibody or circulating antigen detection in serum). The microscopic examination of stool (e.g., the Kato-Katz method and miracidia hatching tests) is considered the “gold standard” for the detection of schistosomiasis[14]. Although the traditional aetiological diagnostic method is sensitive, it is limited in the diagnosis of low-grade and prepatent infections as well as in evaluating drug therapeutic effects. Moreover, the result is interpreted manually and is greatly influenced by the operator's subjectivity. Therefore, it is necessary to establish a method for the diagnosis of schistosomiasis with high sensitivity and specificity.

In this paper, the sensitivity and specificity of the indirect ELISA we established were both 100%, and the results were interpreted electronically. Without human factors, the misdiagnosis rate of this method was reduced. When the aetiology diagnostic method and indirect ELISA method were both used to detect natural infections goats in Nimu County, Tibet, China, the positive detection rate of the indirect ELISA method was higher than that of the aetiological diagnostic method, indicating that the sensitivity of the former method was higher than that of the latter method.

We conclude that indirect ELISA is a promising tool to substantially improve disease surveillance for *S*. *turkestanicum* in livestock. With proper method and guideline development, the ELISA method we established could become an essential component of schistosomiasis control to achieve the goal of elimination in the future.

### Diagnostic techniques

The diagnosis of schistosomiasis turkestanica has been traditionally based on autopsy and egg examination, including faecal washing and sedimentation methods and egg-hatching methods. However, these methods are time consuming, leading to a high misdetection rate and a low recovery rate[15]. In addition, only mature worms can be detected by the diagnostic methods mentioned above; by that time, substantial damage has already occurred due to visceral migration during the acute phase of schistosomiasis turkestanica[5]. In the serological diagnosis of schistosomiasis japonica, the positive rates obtained by IHA and ELISA are similar to those of the miracidia hatching and Kato-Katz faecal examination methods, but they have higher sensitivity than the faecal examination method. Additionally, IHA and ELISA are convenient to perform and suitable for field screening[16]. Both aetiological methods and serodiagnosis are important in the surveillance and prevention of schistosomiasis. Aetiological methods, such as faecal examinations, require the identification of eggs or miracidia with microscopy. Serodiagnosis methods, such as IHA and ELISA, determine antibody levels in the serum. Currently, faecal examination, such as the Kato-Katz method, is still the gold standard for schistosomiasis diagnosis. However, in mass field screenings, many cases may be low-grade infections or prepatent infections, which cannot be easily detected by aetiological methods. The ELISA method reduced the misdetection rate to a certain extent and improved the sensitivity in low-grade infection cases. As a result, ELISA has more advantages than faecal examination in practical applications.

In the ELISA for *S*. *japonica*, which belongs to the same genus as *S*. *turkestanicum*, SEA is usually employed as the diagnostic antigen to improve the sensitivity of the assay[17]. Jiang S F detected schistosomiasis in humans with purified SEA, and the sensitivity of his method reached 93.7%[18]. Zhang et al[19,20] selected 6 proteins with diagnostic value with an antigenic immunoproteomics technique. Two of the proteins, phosphate glycerate mutase (PGM) and radiosensitive protein (RAD23), were chosen and applied in the ELISA. In the detection of buffalo serum positive for Schistosoma, PGM achieved 96% sensitivity and RAD23 achieved 89.33% sensitivity. Considering SEA as the control, the sensitivity reached 100%, indicating a high potential and value of SEA in ELISA.

Thus, further studies on the application of *S*. *turkestanicum* SEA as the diagnostic antigen used in ELISA are worth performing. Therefore, we proposed *S*. *turkestanicum* SEA as a diagnostic antigen.

Due to the lack of eggs spawned by homozygous mature female worms (female *S*. *turkestanicum* worms contain only 1 egg), it is difficult to isolate enough SEA for ELISA application. According to Shi Bin, approximately 73% of *S*. *turkestanicum* eggs deposited in Tibetan goats are distributed in the host intestine [8].

Zhao Dengyun prepared *S*. *japonicum* eggs from liver tissue by the NaOH erosion method, which is fast, simple and high-yielding, and the SEA showed intact antigenicity [21]. In this study, the NaOH erosion method was used to prepare *S*. *turkestanicum* eggs from the small intestine of infected goats. SEA was used as the diagnostic antigen to establish the ELISA testing method, and the sensitivity and specificity of SEA were analysed. Serum samples collected on the spot were tested, and the results were compared with those of faecal examination.

## Materials and methods

### Ethics statement

The goats were purchased in Nimu County, Tibet. This study was carried out in strict accordance with the recommendations in the Animal Ethics Procedures and Guidelines of the People’s Republic of China. Investigation of the egg distribution was performed in 3 goats under 3% sodium pentobarbital anaesthesia (3.9–4.2 ml/kg body weight (BW) or 117–126 mg/kg BW). The eyelid radiation, pupil dilatation, muscle relaxation and heart rate of the anaesthetized goats were strictly monitored. Surgery started only when the goats were confirmed to have died. The protocol was approved by the Committee on the Ethics of Animal Experiments of the Tibet Academy of Agricultural and Animal Husbandry Sciences (Protocol Number: XKKG2017001).

Sera used in this study were collected from goats infected with *S*. *turkestanicum* in local villages in Nimu County, Tibet, with oral consent from the owners. Blood was obtained from neck veins without threatening their lives.

### Reagents

Rabbit anti-goat IgG with HRP, skim milk powder, 1x coating buffer, TMB substrate solution were obtained from Beijing Solebo Co., Ltd.; Tween-20 was obtained from China Pharmaceutical Group Chemical Reagents Co., Ltd.; and BCA protein concentration kit was obtained from Takara Co.

### Serum samples

All serum samples were stored in the laboratory.

Forty-eight goats were naturally infected with *S*. *turkestanicum* according to positive faecal examinations (hatching method) in Nimu County, Tibet, in 2016.

One hundred negative goat serum samples were collected from non-endemic schistosomiasis areas (Shandong Province), and the faecal examination results were negative.

A total of 54 buffaloes were artificially infected with *S*. *japonicum*, with positive faecal examinations (hatching method) in 2015. The serum samples were preserved in the laboratory.

Buffalo sera negative for *S*. *japonicum* was collected from buffaloes that had not been artificially infected, and the faecal examination results were all negative.

### Investigation of egg distribution

In the schistosomiasis turkestanica-endemic area of Tibet, we verified *S*. *turkestanicum*-positive goats by the hatching method. Three goats were randomly selected for necropsy to detect the distribution of *S*. *turkestanicum* eggs in the goats.

After autopsy, the liver, small intestine, large intestine and spleen were isolated. The intestinal contents were flushed with normal saline. The small intestine was separated into 3 parts: the duodenum, jejunum and ileum. The caecum, colon and rectum were also separated. All the samples were weighed and recorded. The NaOH erosion method was applied to obtain eggs from the samples. After observation by microscopy, a certain number of eggs were counted 3 times. Eggs per gram (EPG) values and the percentage egg distribution were determined.

### Collection of adult worms and eggs

Positive goats were identified by faecal examination. The thoracic cavity was opened, and the adult worms were collected by perfusion. The adult worms were washed repeatedly with PBS and subsequently stored in liquid nitrogen.

Over 60% of the eggs of *S*. *turkestanicum* were distributed in the small intestine of Tibetan goats[8]. In the samples consisting of the duodenum to the end of the small intestine in positive goats, we isolated the eggs of *S*. *turkestanicum* by the NaOH erosion method and washed them with PBS. After observation under microscopy, the collected eggs were stored in liquid nitrogen for future use.

### Preparation of soluble antigen from adult worms and eggs

Referring to the study[21] by Zhao Deng-yun, adult worms or eggs were placed in a macerator with a proper amount of saline (0.9% sodium chloride) and repeatedly macerated at a low temperature until no particles were visible to the naked eye. The fluid was then collected and subjected to freezing and thawing three times. The fluid was then ultrasonicated for 20 min at 900 W, with 2 sec on and 9 sec off. The supernatant was finally obtained by centrifugation at 1,500 ×g for 5 min, as were the SWAP and SEA[21]. The protein concentration was measured by the bicinchoninic acid (BCA) method.

### Optimal concentration of antigen coating, determination of serum dilution

SWAP and SEA were diluted to 0.5 μg/ml, 0.75 μg/ml, 1.0 μg/ml, and 1.5 μg/ml. A plate was coated with 100 μl of diluted antigens per well and stored overnight at 4°C. The plate was then washed twice with 1% Tween in PBS (PBST). A 5% skim milk solution was added to the plate (200 μl/well), and the plate was incubated at 37°C for 1 hour. The plate was washed three times with PBST. The standard negative and positive sera were diluted to produce 1:50, 1:100, 1:150 and 1:200 concentrations to establish a square array experiment. The corresponding serum dilution was added to each antigen solution. A serum diluent was added to each well, followed by incubation for 1 hour at 37°C. Each well was then washed 3 times with PBST; the rabbit anti-goat antibody labelled with enzyme was diluted to a 1:4000 concentration. The reaction complex was washed 3 times with PBST. After washing, soluble double-component TMB substrate was supplied for the reaction, and the reaction was subsequently quenched with 2 M H_2_SO_4_ added at 50 μl per well. The absorbance was measured at 450 nm.

### Sensitivity and specificity of ELISA

In light of the optimal ELISA conditions determined in previous experiments, *S*. *turkestanicum*-positive sera from 48 goats and *S*. *turkestanicum*-negative sera from 100 goats were examined by ELISA. The sensitivity and specificity of SEA were analysed.

### Test for an antigenicity crossover reaction between the *S*. *turkestanicum* egg antigen and *S*. *japonicum* egg antigen

According to the determined experimental conditions, the concentration of the antigen coating was 0.5 μg/ml, the blocking solution contained 5% skim milk powder, the best concentration for serum diluents was 1:100, and the serum diluent contained 5% skim milk powder. The other steps were the same as those mentioned previously. Fifty-four buffaloes that presented hatching *S*. *japonicum* miracidia in their stool were tested. The negative and positive serum control groups were tested simultaneously. The optical density at 450 nm (OD_450nm_) was measured, and cross-reactivity was determined according to critical values between negative and positive samples.

### Detection of clinical samples

Detection was conducted in a natural epidemic area of schistosomiasis turkestanica in Nimu County, Tibet Autonomous Region. The faecal hatching test in random goats showed that the infection rate of local goats was 72%. A systematic serological investigation of goats in this area was carried out. A total of 114 serum samples were collected and tested for schistosomiasis turkestanica by the indirect ELISA diagnostic method. Goats were grouped according to their ages to analyse *S*. *turkestanicum* infection in different age groups.

### Statistical analysis

SPSS 16.0 statistical software was used, and P < 0.05 was considered statistically significant. One-way ANOVA was used to determine the statistical significance of the differences among groups. Each individual experiment was conducted with at least 3 goats, and each experiment was repeated at least twice.

## Results

### Distribution of *S*. *turkestanicum* eggs in different host organs

**Fig 1:**
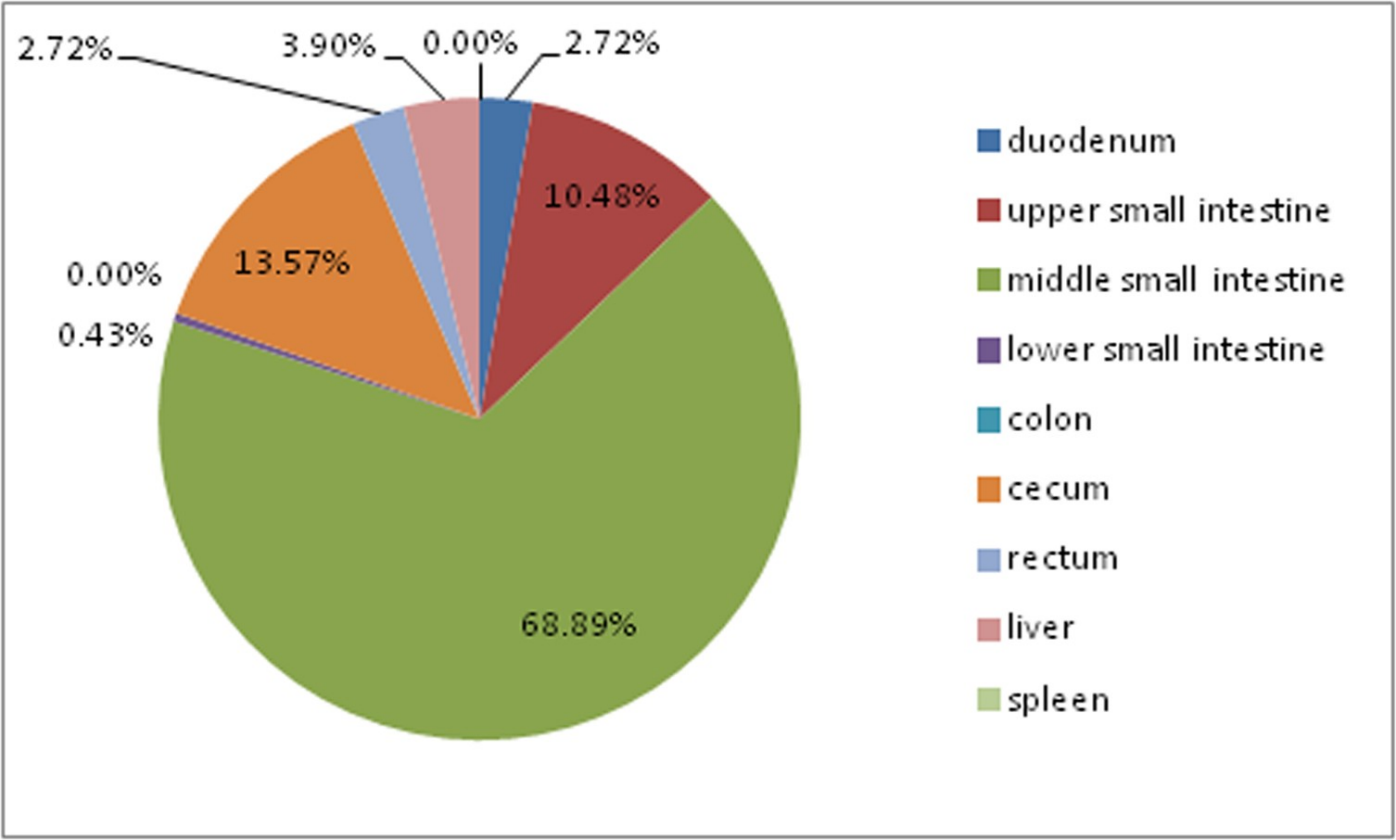
Distribution of *S*. *turkestanicum* eggs in different organs in Nimu goats. The majority of the eggs were distributed in the host small intestine. Subdividing the small intestine into three parts (upper, middle, lower), the middle part harboured the majority of the egg burden (68.89%), followed by the caecum (13.57%) and upper small intestine (10.48%). Approximately 2.72% of the eggs were found in the duodenum. There were low numbers of eggs in the lower small intestine (0.43%), colon (0.00%) and rectum (0.01%). Very few eggs were found in the liver (3.90%) and spleen (0.00%).

Faecal examinations were conducted to identify infected goats. A total of 3287 worms were collected from the host mesenteric vein and hepatic portal vein by aorta abdominalis perfusion. The provided pie charts (Fig 1) show a breakdown of the distribution of the *S*. *turkestanicum* eggs in host organs. The results were consistent with a previous study conducted by Shi Bin [8].

### Preparation of SWAP and SEA

**Fig 2:**
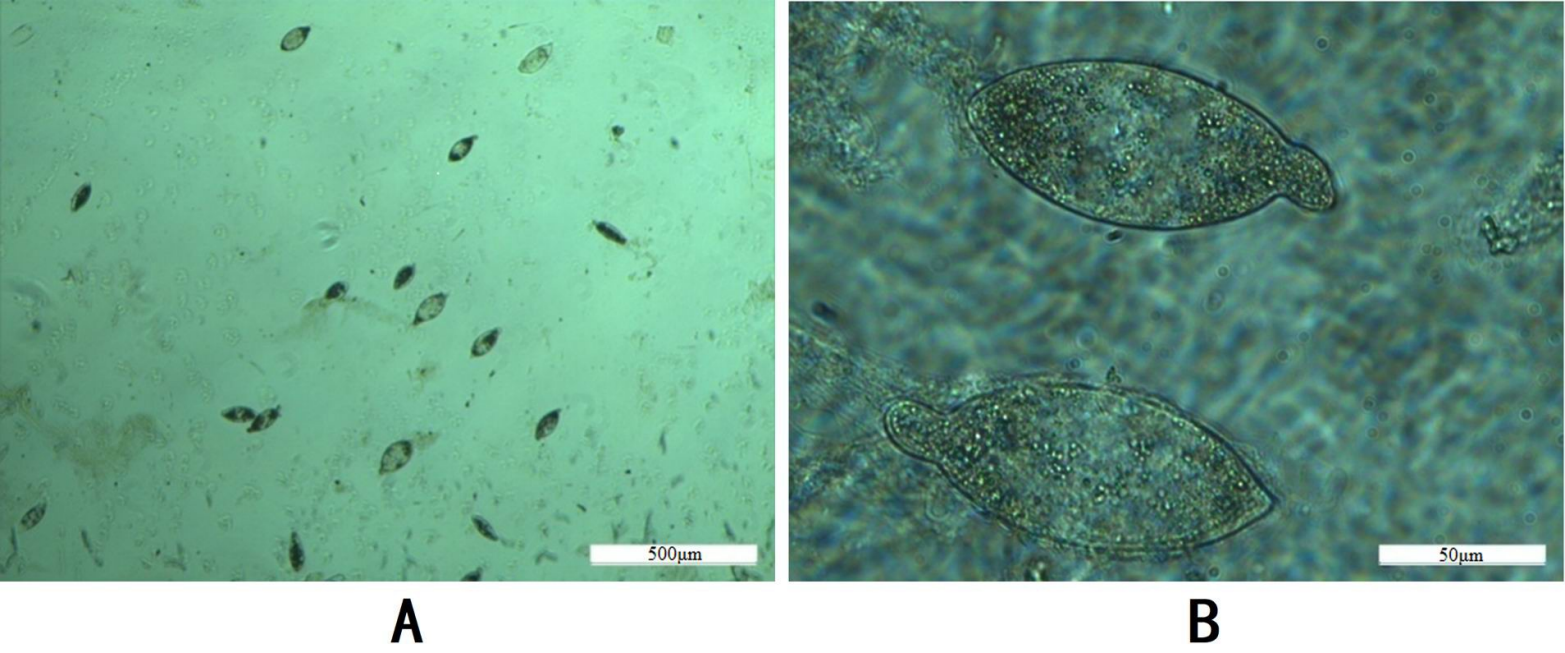
*S*. *turkestanicum* eggs. A: Image of the collected eggs using the NaOH erosion method; B: Structure of the eggs.

*S*. *turkestanicum* eggs were oval in shape, and the eggshell exhibited a characteristic structure with an apical projection on the surface. The eggshells of the *S*. *turkestanicum* eggs were intact, as was the internal shape of the eggs. The miracidia were clear and discernible. No adherent tissue fragments conglutinated to the eggshell or other impurities were observed, as shown in Fig 2. The concentration of SEA was 0.9118 mg/ml, and that of SWAP was 0.6825 mg/ml.

### Determination of the best concentrations for the coated antigens and serum dilution

Considering P/N = 2.1 as the threshold, P/N≥ 2.1 was considered to indicate positive results. The best concentrations of the sera and coated antigens were fixed at 1:100 and 0.5 μg/ml, respectively, by a square titration experiment. The results, shown in Table 1 and Table 2, indicated that 5% skim milk powder was the best blocking and serum diluent agent.

**Table 1:** Best concentrations of sera and coated SEA.

Concentration of SEA (μg/ml)	Positive serum dilution				Negative serum dilution				P/N			
	200	150	100	50	200	150	100	50	200	150	100	50
0.5	0.5482	0.6776	0.6470	0.6609	0.0918	0.1138	0.1084	0.1296	5.9744	5.9569	5.9686	5.1011
0.75	0.4587	0.4903	0.5409	0.6498	0.0941	0.0945	0.1079	0.1266	4.8772	5.1911	5.0153	5.1315
1.0	0.4619	0.5115	0.5724	0.7256	0.0948	0.1107	0.1027	0.1408	4.8724	4.6206	5.5730	5.1534
1.5	0.3615	0.3547	0.4378	0.5749	0.0817	0.0878	0.0851	0.0897	4.4268	4.0399	5.1476	6.4127

**Table 2:** Best concentrations of sera and coated SWAP.

Concentration of SWAP (μg/ml)	Positive serum dilution				Negative serum dilution				P/N			
	200	150	100	50	200	150	100	50	200	150	100	50
0.5	0.1356	0.1527	0.1778	0.2086	0.0777	0.0757	0.0759	0.0844	1.7434	2.0172	2.3426	2.4701
0.75	0.1590	0.1677	0.1666	0.1935	0.0747	0.0718	0.0796	0.0842	2.1285	2.3373	2.6936	2.2987
1.0	0.1980	0.2029	0.2350	0.2778	0.0745	0.0733	0.0814	0.0882	2.6563	2.7683	2.8870	3.1494
1.5	0.2228	0.2661	0.2844	0.3223	0.0766	0.0734	0.0825	0.0815	2.9105	3.6247	3.4500	3.9564

### Sensitivity, specificity, and cross-reactivity of the method

**Fig 3:**
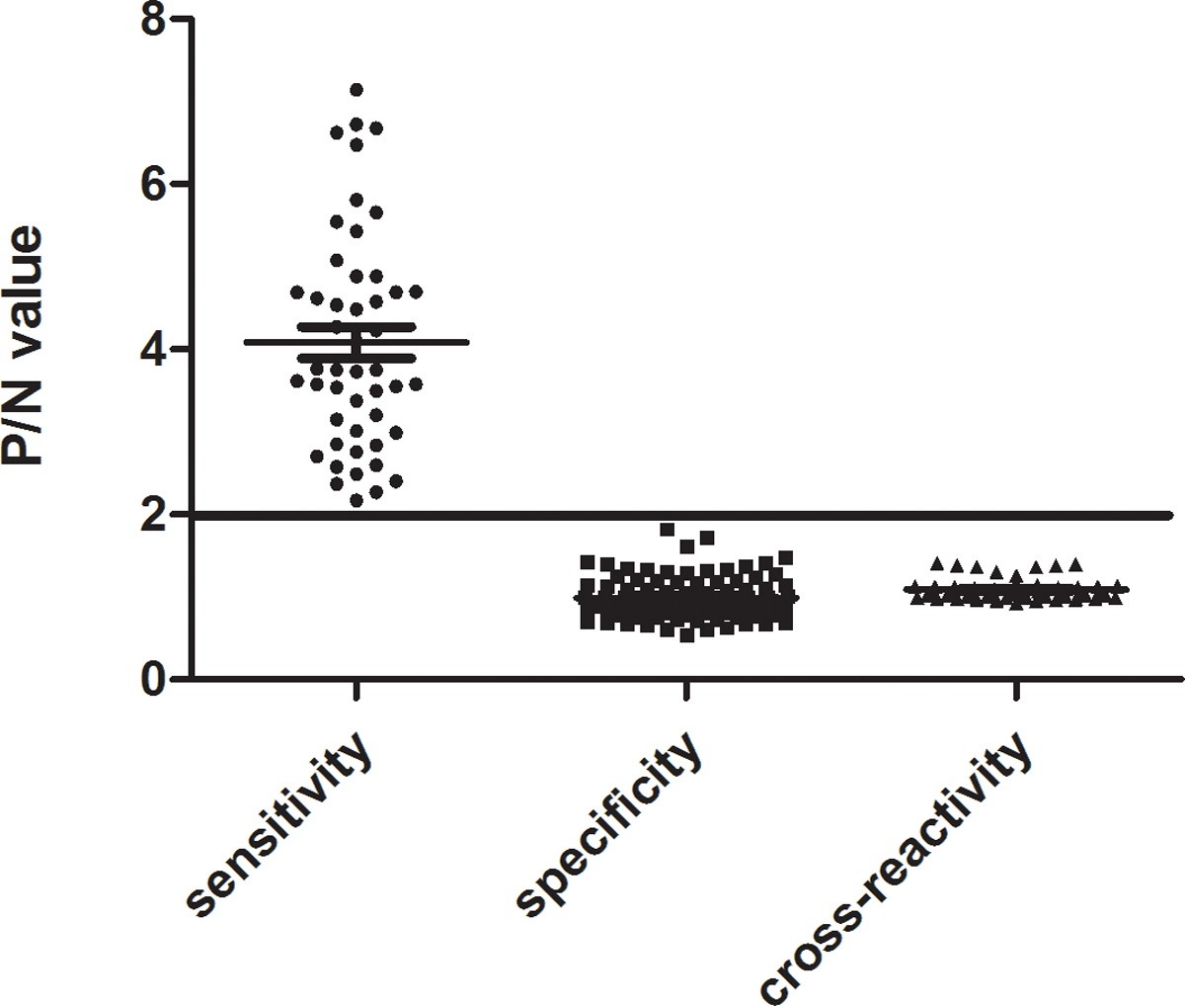
Specificity, sensitivity and cross-reactivity detection. Each OD value is representative of the mean of three absorbance values. The sensitivity and specificity testing groups comprised 100 goat sera samples negative for *S*. *turkestanicum* and 48 goat sera samples positive for *S*. *turkestanicum*. The cross-reactivity group comprised 54 buffalo sera samples positive for *S*. *japonicum*. The charts were created in Prism 5.0 software.

Considering P/N ≥ 2.1 as the threshold and conducting the experiments indicated above, 100 specimens of negative sera and 48 goats that presented hatching *S*. *turkestanicum* miracidia in their stool were analysed by indirect ELISA, with SEA as the diagnostic antigen. The specificity and sensitivity were determined, and the original data was shown in S1 Table. Fifty-four buffaloes that presented hatching *S*. *japonicum* miracidia in their stool were tested for cross-reactivity. The data are shown in Fig 3.

Forty-eight positive goat serum samples (confirmed by the faecal hatching test) were diluted 100 times, and the P/N values were all appropriate for the positive standard. The sensitivity of the ELISA established in this experiment was 100%.

One hundred negative goat (confirmed by the faecal hatching test) sera samples were diluted 100 times and tested, and all of them were found to be negative. The specificity of the ELISA established in this experiment was 100%.

The cross-reactivity test showed that the P/N values of serum samples from 54 goats infected with schistosomiasis japonica did not reach those of the positive standard; therefore, there was no cross-reactivity with schistosomiasis japonica.

### Clinical sample detection

**Fig 4:**
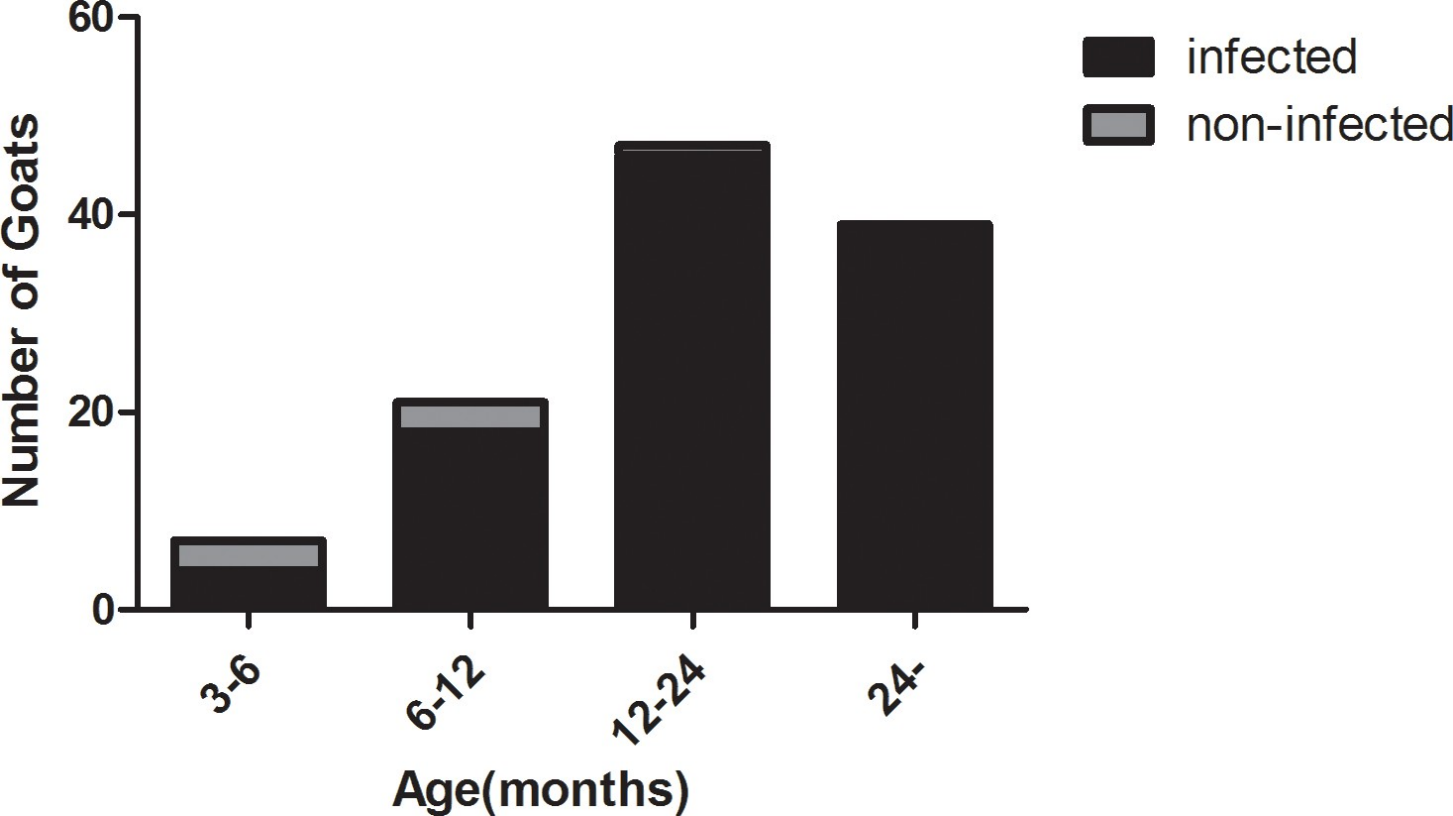
Clinical sample detection. 3-6: There were 7 goats aged from 3 to 6 months in this group, 4 of which were infected with *S*. *turkestanicum* according to the ELISA; 6–12: There were 21 goats aged from 6 to 12 months in this group, 18 of which were infected with *S*. *turkestanicum* according to the ELISA; 12–24: There were 47 goats aged from 12 to 24 months in this group, 46 of which were infected with *S*. *turkestanicum* according to the ELISA; 24-: There were 39 goats aged over 24 months in this group, 39 of which were infected with *S*. *turkestanicum* according to the ELISA.

ELISAs was carried out in 114 grazing goats in a natural village in the schistosomiasis turkestanica epidemic area. The overall infection rate was 93%. The positive rate of the serum samples according to the ELISA was higher than that according to the faecal examination method (72%). The data from these serological tests were stratified according to age of the goats. The data are shown in Fig 4. The positive rate of goat infection in the epidemic area increased gradually with age, and the infection rates were 57.14%, 85.71%, 97.87% and 100%.

## Discussion

Schistosomiasis turkestanic a is a parasitic disease that is prevalent in livestock, with parasitization mainly occurring in the portal vein and mesenteric veins of mammals, such as cattle or goats. *S*. *turkestanicum* survives by ingesting nutrients from sources such as the host's red blood cells and reproduces sexually in the host. Excreted eggs often cause congestion and swelling in the duodenal mucosa of the small intestine, clear lesions on the intestinal wall, milky nodules and mottled tissue[22]. This disease represents a great threat to the health of infected animals, often leading to their eventual debilitation and death. The disease is widely distributed in China[5]. Its intermediate hosts are species such as *Radix auricularia*, *Radix ovata*, *Radix lagotis* and *Radix peregra*[23]. The disease mainly occurs in low-lying, humid, rainfall-rich areas. The intermediate hosts mainly inhabit shallow waters, such as paddy fields, river ponds and swamps. Therefore, the prevalence of schistosomiasis turkestanica is also related to the season and the timing and amount of annual rainfall. The earlier the rainfall is, the more serious the disease will be[24]. After the 1998 floods in northeast China, the epidemic area of the disease expanded. In 1999, up to 1.8 million livestock animals were infected in Jilin, Tibet, Heilongjiang and Inner Mongolia, causing substantial economic losses[23]. The detection of *S*. *turkestanicum* was an important step in controlling the disease.

*S*. *turkestanicum* and *S*. *japonicum* belong to the same genus and exhibit a close relationship. The SEA of *S*. *japonicum* is mainly used as a diagnostic antigen. Therefore, the possibility of using soluble antigens of *S*. *turkestanicum* eggs as diagnostic antigens was studied by referring to the detection of *S*. *japonicum*.

Eggs were mainly distributed in the intestinal wall of the host, especially in the duodenum and small intestine. There is more muscle and keratinized tissue in the intestinal wall, and the homogenate is difficult to break down. The method for purifying *S*. *japonicum* SEA was not suitable for *S*. *turkestanicum* because the muscle and keratinized tissue were likely to block the sieve. Therefore, the mucosal layer of the intestinal wall was scraped and eroded by NaOH. This step not only avoided the loss of eggs during the screening process but also increased the egg yield. The mucosal tissue was easily liquefied in a strong alkali solution. The eggs have a layer of eggshell that protects the inner tissue from strong alkalinity. The obtained eggs were pure and intact. Materials for extracting eggs were easily prepared by centrifugation and washing. The method was fast and suitable for extracting eggs of *S*. *turkestanicum*, which are difficult to obtain using traditional methods.

In this study, a comparison of the diagnostic effect of SEA and SWAP was conducted. When the same amounts of coated antigen and serum dilutions were used, the P/N value of SEA was much higher than that of SWAP. The P/N values for different concentrations of SEA were all above 4.5, while those for SWAP with different coating concentrations varied greatly. Moreover, SWAP exhibited indistinct P/N values (approximately 2) at particular coating concentrations. As a diagnostic antigen for schistosomiasis turkestanica ELISA detection, SEA was more appropriate than SWAP.

The reason for this result may be that there is a series of immune evasion steps in adult worms, and the specific antibodies induced by SWAP in the host body may therefore be insufficient or absent. The life cycle of the eggs is simple; they stimulate the immune response of the host body to induce inflammation, thereby penetrating the intestinal wall tissue and entering the intestinal cavity. Therefore, the specific antibody levels produced are high. The ELISA detection method in which SEA was applied as the diagnostic antigen was more effective than that with SWAP.

To reduce the non-specificity of ELISA, we manipulated the serum diluent, dilution ratio, coating concentration and blocking solution. Eventually, we established an indirect ELISA method using SEA as the diagnostic antigen for schistosomiasis turkestanica detection. Both the sensitivity and specificity of the method were good, which verified that positive sera and negative sera could be clearly distinguished. Moreover, the method showed no cross-reactivity with *S*. *japonicum*.

The field epidemiological analysis indicated a relationship between the *S*. *turkestanicum* infection rate and the age of the goats. Young goats exhibited a decreased infection rate. The reasons for this relationship might involve two factors: 1) the low chance of contact with contaminated water among young goats, especially in the lactating or suckling period; and 2) the immaturity of the parasites, which can avoid detection. Goats over 1 year old showed an approximately 100% infection rate according to the ELISA; this rate was higher than that according to aetiological detection (faecal hatching), suggesting a difference in the sensitivity of the two methods. The sensitivity of the faecal hatching method was low, and the possibility of missed detection existed, while the sensitivity of serological detection was high, and the rate of missed detection was decreased. Considering the rate of missed detection, serological detection (ELISA) data are recommended.

*S*. *turkestanicum* was found to be endemic and prevalent. Although animal husbandry is seriously impacted and cercariae-induced dermatitis threatens human health, the understanding of the disease is still insufficient, and the preventative and treatment measures for the disease are still weak. The infection rate reported in our field epidemiological analysis was high. Considering these findings combined with the high intensity of infection, the state of local schistosomiasis turkestanica infection is already very serious and should receive substantial attention. According to the characteristics of *S*. *turkestanicum*, a set of measures for the diagnosis, treatment, prevention and control should be established to control the epidemic of the disease as soon as possible.

The indirect ELISA for schistosomiasis turkestanica detection required 0.5 μg/ml coating antigen and a 1:100 serum dilution ratio. The data were acquired using a microplate reader. The results were reliable, and the method was suitable for the large-scale detection of *S*. *turkestanicum*.

## Supporting information

S1 Table The original data(OD_450_)of the sensitivity, specificity and cross-reactivity detection. (XLSX)

## Abbreviations

ELISA: Enzyme-linked immunosorbent assay
*S. japonicum*: *Schistosoma japonicum*
*S. turkestanicum*: *Schistosoma turkestanicum*
IHA: Indirect hemagglutination assay
SEA: Soluble egg antigen
NaOH: sodium hydroxide
PBST: 1% Tween in PBS
